# Lab-scale production of postbiotic proteins from *Bifidobacterium adolescentis* with antiviral and epithelial-protective properties

**DOI:** 10.3389/fmicb.2025.1646082

**Published:** 2025-10-01

**Authors:** María Hernández, Balkys Quevedo, María Cabrera, Juan Ulloa

**Affiliations:** ^1^Grupo de Enfermedades Infecciosas, Departamento de Microbiología, Facultad de Ciencias, Pontificia Universidad Javeriana, Bogotá, Colombia; ^2^Grupo de Biotecnología Ambiental e Industrial (GBAI), Departamento de Microbiología, Facultad de Ciencias, Pontificia Universidad Javeriana, Bogotá, Colombia

**Keywords:** *Bifidobacterium adolescentis*, postbiotics, bioreactor, rotavirus, gut epithelium, antiviral activity, cytoskeletal integrity, functional ingredients

## Abstract

Postbiotics produced by probiotic bacteria are gaining attention as multifunctional, food-derived agents with potential applications in human and animal health. This study investigates the production and biological activity of protein-rich postbiotics from *Bifidobacterium adolescentis*, cultivated under controlled conditions in a 3-liter bioreactor as a laboratory-scale model for functional ingredient development. Culture parameters were improved, and one representative batch was selected for biological evaluation. The postbiotic preparation was tested for cytotoxicity using MA104 (renal) and C2BBe1 (intestinal) epithelial cell lines through viability and cell death assays, confirming its safety across a range of concentrations. To assess its functional activity, we evaluated its ability to reduce rotavirus infection and preserve epithelial integrity. The postbiotic significantly reduced viral infectivity and maintained cytoskeletal architecture in infected intestinal cells, supporting its potential protective role. These findings suggest that *B. adolescentis*-derived postbiotics may serve as safe and biologically active compounds with potential applications in intestinal health and viral infection management.

## Introduction

A postbiotic is defined as “a preparation of inanimate microorganisms and/or their components that confers a health benefit on the host” (Salminen et al., 2021). These preparations may include microbial-derived substances such as metabolites, cell wall components, or proteins (Thorakkattu et al., 2022; Vinderola et al., 2022). For a substance to be classified as a postbiotic, it must originate from a well-characterized microorganism with a fully sequenced genome and be produced through a well-defined, reproducible process of biomass generation and inactivation (Vinderola et al., 2022). The growing interest in functional foods, including probiotics and postbiotics, stems from their potential to deliver health benefits through bioactive compounds (Wegh et al., 2019; Thorakkattu et al., 2022; Zhong et al., 2022; Szajewska and Salminen, 2023). Scientific evidence suggests that postbiotics may enhance immune function, improve digestive health, and support the management of gastrointestinal disorders (Thorakkattu et al., 2022).

Diarrheal disease is a major global health concern and remains one of the leading causes of morbidity and mortality in children under 5 years of age, accounting for approximately 9% of deaths in this age group, with an estimated 443,832 deaths annually (UNICEF, 2023; WHO, 2024). While dehydration has historically been the primary cause of diarrhea-related mortality, bacterial infections and sepsis are increasingly recognized as significant contributors. Malnourished and immunocompromised children, including those living with HIV, are at highest risk. Standard treatment includes oral rehydration salts (ORS) and zinc supplementation to restore fluids, reduce illness duration, and lower mortality risk (WHO, 2024).

Rotavirus (RV) is the leading cause of acute diarrheal disease (ADD) in children, with infection rates in hospitalized cases ranging from 30% to 50% worldwide (Omatola and Olaniran, 2022; Sadiq and Khan, 2023; Omatola et al., 2024). This non-enveloped virus, part of the Reoviridae family, has a segmented double-stranded RNA genome encoding structural and non-structural proteins. RV primarily spreads via the fecal–oral route and infects mature enterocytes and enteroendocrine cells in the small intestine, leading to epithelial damage and disruption of intestinal homeostasis. NSP4, one of its key virulence factors, contributes to pathogenesis by altering intracellular calcium signaling, impairing transporters, disrupting the cytoskeleton, and compromising tight junction integrity (Desselberger, 2014; Omatola and Olaniran, 2022).

Despite the global deployment of vaccines such as RotaTeq^®^ and Rotarix^®^ since 2006, and more recently Rotasiil^®^ and Rotavac^®^, vaccine efficacy remains suboptimal in high-burden regions, ranging from 50% to 66%, with limited protection against emerging or region-specific strains (Armah et al., 2010; Zaman et al., 2010; Madhi et al., 2016; Donato et al., 2021). Oral rehydration therapy (ORT) continues to be the primary treatment, addressing dehydration but not the viral infection itself. To date, there is no approved antiviral targeting RV or other enteropathogenic viruses, highlighting a critical gap in therapeutic options (Omatola and Olaniran, 2022).

Probiotics have been studied for over three decades as supportive therapies for acute diarrheal disease (ADD), including cases caused by rotavirus (RV), particularly in infants and young children (Allen et al., 2010; Erdoğan et al., 2012; Iqbal et al., 2014). Multiple clinical studies have demonstrated that specific probiotic strains–especially those from the genera *Lactobacillus* and *Bifidobacterium*–can reduce both the duration and severity of diarrheal episodes (Allen et al., 2010). These effects are primarily attributed to the ability of probiotics to modulate the host immune response, enhance mucosal defenses, and promote a balanced intestinal microbiota (Latif et al., 2023).

While live probiotics have shown clinical benefit, their limitations in terms of viability, safety, and standardization have led to growing interest in alternative or complementary approaches. Among these, bioactive compounds derived from probiotic bacteria–commonly referred to as postbiotics–have gained attention as novel adjunct therapies. *Bifidobacterium* strains, particularly *Bifidobacterium adolescentis*, have demonstrated antiviral activity against RV in both animal models and human cell lines (Muñoz et al., 2011; Kang et al., 2015). *In vitro* studies have shown that metabolites from *B. adolescentis* can reduce RV infectivity by up to 50.99% and decrease intracellular levels of NSP4 by approximately 20%, suggesting a dual mechanism of action involving both inhibition of viral replication and attenuation of cytotoxicity (Olaya Galán et al., 2016; Fernandez-Duarte et al., 2018). These findings position *B. adolescentis* metabolites as promising candidates for adjunct therapeutic strategies in the management of viral diarrhea.

Postbiotics offer an attractive alternative to live probiotics due to their improved safety profile and stability. However, the production of postbiotics presents important challenges, particularly regarding standardization, scalability, and preservation of bioactivity. Factors such as culture conditions–temperature, pH, oxygen levels, and agitation–can influence both bacterial growth and the quality of the bioactive compounds produced (Cheigh et al., 2002; Grześkowiak et al., 2011). Consistent quality, safety, and reproducibility across batches are essential for regulatory approval and commercial use (Ouwehand et al., 2018; Cano-Lozano et al., 2022). Additionally, maintaining stability during storage, identifying the active components, and scaling up production without compromising efficacy remain key hurdles. These challenges underscore the need for well-defined bioprocesses to ensure the reliable production of clinically effective postbiotics.

In 2023, we reported the evaluation of proteins secreted by *Bifidobacterium adolescentis* (BaSP), obtained from flask-grown cultures, and demonstrated that these proteins exhibit direct antiviral activity by significantly reducing rotavirus (RV) infectivity in human intestinal C2BBe1 cells. Additionally, BaSP preserved epithelial barrier integrity, as evidenced by the maintenance of transepithelial electrical resistance (TEER) and the expression of occludin, a key tight junction protein (Hernández et al., 2023). These findings suggest that BaSP exert dual functionality by reducing viral infectivity and reinforcing epithelial barrier integrity. However, advancing their use in food or therapeutic applications requires verifying whether these bioactivities are retained under scalable and controlled production systems such as bioreactors. It is also essential to explore the mechanisms through which these postbiotics exert structural protection–particularly their potential role in preserving cytoskeletal integrity, which is often compromised during RV infection. In this context, the present study evaluated the *in vitro* safety, antiviral activity against rotavirus, and impact on cytoskeletal architecture of bioreactor-produced postbiotics from *Bifidobacterium adolescentis* (BaP). The study also supports the development of scalable, controlled bioproduction systems for the generation of postbiotic-based therapeutic strategies targeting gastrointestinal viral diseases.

## Materials and methods

### Activation of *Bifidobacterium adolescentis*

Lyophilized *Bifidobacterium adolescentis* (DSMZ GmbH, Braunschweig, Germany) was reactivated in de Man, Rogosa and Sharpe (MRS) broth (Oxoid, Basingstoke, UK) under anaerobic conditions at 37 °C using an anaerobic jar with gas generation system (AnaeroGen™, Oxoid). Purity was confirmed using the BBL™ Crystal™ Enteric/Non-fermenter ID Kit (Becton, Dickinson and Company, Franklin Lakes, NJ, USA), and viability was determined to be ≥107 CFU/mL.

### Improvement of culture conditions in lab-scale bioreactor

To improve biomass production, agitation speed, inoculum concentration, and medium strength were evaluated. Microcultures (5 and 25 mL) were incubated at 37 °C under anaerobic conditions with different agitation speeds (0, 50, 100, and 150 rpm), two inoculum concentrations (5% and 10% v/v), and MRS dilutions (undiluted MRS medium, 1:2, and 1:5). Biomass was quantified every 6 h by dry weight as described by Pedroza-Rodríguez et al. (2007). All experiments were performed in triplicate in three independent assays (Pedroza-Rodríguez et al., 2007). The calibration curve correlating absorbance with biomass dry weight (g/L) can be seen in the (Supplementary Figure 1).

Controlled fermentation was conducted in a 3 L Applikon bioreactor (Applikon Biotechnology, Delft, Netherlands) with a 1.5 L working volume. The system was equipped with dual Rushton turbines, rotameters for CO_2_ and N_2_ dosing, and integrated pH and DO sensors. Cultures were maintained at 37 °C, with continuous injection of N_2_ and CO_2_ to maintain anaerobic conditions and pH stability. Dissolved oxygen was kept at 0%, and pH at 6.8 ± 0.1, automatically regulated using 2N NaOH and 2N HCl. Agitation was set to 200 rpm to improve CO_2_ dispersion. Samples were taken every 6 h for biomass and protein analysis.

### Production of postbiotic proteins

Three bioreactor batches (1.5 L each) were harvested at the end of exponential phase. Cultures were centrifuged at 7,500 × *g* for 30 min at 4 °C. Supernatants were ultrafiltered with a Pellicon^®^ XL 50 cassette (Merck Millipore), and polyethylene glycol (PEG) 8000 (10% w/v) was added for protein precipitation. The mixture was stirred overnight at 4 °C and centrifuged at 16,100 × *g* for 30 min. Pellets were resuspended in sterile PBS (1X) and filtered through 0.22 μm membranes. BaP was stored at 4 °C in sterile PBS until use.

BaP refers to postbiotics proteins derived from bioreactor cultures, while BaSP refers to those obtained from flask cultures, as previously described. This distinction is consistently maintained throughout the manuscript.

Two control preparations were included: (1) an abiotic control (AC), consisting of sterile MRS broth subjected to the same downstream processing as the experimental samples; and (2) a production control (PC), obtained by culturing *B. adolescentis* in 500 mL flasks under anaerobic, static conditions at 37 °C for 30 h, as previously described by Hernández et al. (2023). Both underwent the same protein isolation process. Protein content was determined using the BCA Protein Assay Kit (Thermo Scientific™, Cat #23227) with BSA as standard. Protein integrity was assessed by native PAGE (7.5%) followed by silver staining.

### Cell lines and virus

MA104 (African green monkey kidney epithelial cells, ATCC CRL-2378.1) and C2BBe1 (a homogeneous subclone of human colon adenocarcinoma Caco-2 cells, ATCC CRL-2102) were used in different stages of the study based on their established functional roles. MA104 cells were primarily employed for antiviral assays due to their high susceptibility to rotavirus infection and are considered the standard model for rotavirus propagation and titration. In contrast, C2BBe1 cells were selected for cytotoxicity and post-infection structural analyses because of their capacity to differentiate into polarized monolayers that mimic mature enterocytes, including tight junction formation and microvilli expression.

Both cell lines (seeding at 10,000 and 30,000 cells/well respectively) were cultured at 37 °C and 5% CO_2_ in Advanced DMEM (Invitrogen™), supplemented with 5% fetal bovine serum (FBS), 2 mM L-glutamine, and a 1X antibiotic–antimycotic mix (Gibco™). Cells were maintained under standard conditions and used between passages 20 and 30.

For the rotavirus assays, strain RRV (simian rotavirus) was activated with 10 μg/mL of trypsin (Sigma-Aldrich) at 37 °C for 1 h and subsequently inactivated with soybean trypsin inhibitor (Life Technologies™). Two virus stocks were prepared separatelyv by infecting MA104 and C2BBe1 cells, respectively, to account for cell line–specific differences in infectivity and propagation. Viral titers were determined by colorimetric immunocytochemistry using an anti-rotavirus polyclonal antibody and developed with a carbazole-based substrate. The resulting infectious dose was expressed as focus-forming units per milliliter (FFU: focus forming units), and a standardized inoculum of 20,000.

### Cytotoxicity assessment

#### MTT assay

MA104 and C2BBe1 cells were seeded in 96-well plates (10,000 and 30,000 cells/well). After 48 h incubation at 37 °C and 5% CO_2_, cells were treated for 24 h with 14 serially diluted concentrations of BaP (1:2 dilution series), ranging from 250 to 0.03 μg/mL, prepared in sterile 1 × PBS. Parallel control groups included untreated cells (cell control, CC) and cells exposed to 1 mM H_2_O_2_ (cytotoxicity control). Viability was measured with MTT assay (Sigma-Aldrich^®^, Cat #M5655), and absorbance read at 540 nm. The Maximum Non-Toxic Concentration (MNTC) was defined as ≥95% viability.

For repeated-dose testing, cells were exposed to MNTC every 48 h for 14 days. At each time point, the entire culture medium was removed and replaced with fresh medium containing BaP at the MNTC, to ensure continuous exposure and nutrient renewal. Controls included untreated cells and H_2_O_2_-treated cells (1 mM for MA104, 50 mM for C2BBe1). Viability was measured with MTT assay (Sigma-Aldrich^®^, Cat #M5655), and absorbance read at 540 nm. The microplate reader used was the Multiskan™ FC (Thermo Fisher Scientific, USA).

For both single- and repeated-dose assays, cells were treated at ∼80% confluence. During BaP exposure, cultures were maintained in serum-free Advanced DMEM, whose enriched formulation supports long-term monolayer survival, allowing viability assessment over 14 days without passaging.

Each experiment was performed in triplicate and repeated independently three times. Statistical analysis was conducted using the Mann–Whitney U test for independent samples (*p* < 0.05; GraphPad Prism 6.0b).

#### Apoptosis/necrosis markers

Apoptotic and necrotic responses were assessed after 14-day exposure to BaP (250 μg/mL) using the ApoDETECT™ Annexin V-FITC kit (Invitrogen™). Untreated cells incubated in serum-free medium were used as negative controls to establish baseline viability and staining background for Annexin V-FITC and PI detection. Cells were fixed with 2% paraformaldehyde and fluorescence measured in black 96-well plates using a FLUOstar^®^ Omega plate reader. Each experiment was run in triplicate and repeated independently three times. Differences were analyzed using Student’s *t*-test for independent samples (*p* < 0.05; GraphPad Prism 6.0b).

### Virucidal co-incubation assay of BaP against rotavirus

MA104 cells were seeded in black-walled 96-well plates (Costar^®^, Cat# 3603) and cultured for 48 h until reaching ≥95% confluence. BaP (at the MNTC and a 1:2 dilution) was co-incubated at a 1:1 ratio with 20,000 FFU of RRV and subsequently applied to the confluent MA104 monolayers. After 1 h of incubation at 37 °C, the cells were washed with PBS to remove unbound virus and incubated for 9 h in serum-free medium. Cells infected with rotavirus without BaP served as positive infection controls. Uninfected cells without BaP were used as negative controls to define baseline fluorescence and background signal. Cells were fixed with 80% acetone-PBS (v/v) 15 min at RT, washed with PBS. Anti-TLP antibody was added (50 μL, 2 h, RT), followed by Alexa Fluor™ 488-conjugated goat anti-rabbit IgG (50 μL, 1 h, RT). Fluorescence was read at 488/520 nm. Data were from three independent experiments, triplicates. Fluorescent foci were imaged with Olympus CKX-41 microscope (Olympus Corporation, Tokyo, Japan) equipped with Lumin Epi-Fluorescence Module. Statistics: Student’s *t*-test (*p* < 0.05).

### Post-infection effect of BaP on C2BBe1 architecture

C2BBe1 cells were seeded on 6 mm glass coverslips in 24-well plates and cultured until reaching ≥95% confluence. For post-infection treatment assays, cells were infected with 20,000 FFU/well of trypsin-activated rotavirus strain RRV and incubated for 1 h at 37 °C to allow viral adsorption. Afterward, cells were gently washed three times with sterile PBS to remove unbound viral particles and incubated in serum-free medium. At 3 h post-infection (hpi), BaP was added to the designated treatment wells. RV-infected cells without BaP treatment served as positive controls for virus-induced cytoskeletal disruption, while uninfected cells in serum-free medium were used as negative controls. Cells were fixed at 6 and 9 hpi using 2% paraformaldehyde for 20 min at room temperature. Permeabilization was performed with 0.03% Triton™ X-100 for 5 min. Actin filaments were stained using Alexa Fluor^®^ 488-phalloidin (Molecular Probes™, Invitrogen™, Cat# 12379) for 30 min at room temperature, and nuclei were counterstained with DAPI (Thermo Fisher Scientific). Coverslips were mounted in 50% glycerol in PBS and visualized using a Zeiss HAL 100 epifluorescence microscope (Zeiss, Oberkochen, Germany). Images were processed using Zen 2.3 lite, FotoCanvas v1.1, and ImageJ software. The experiment was performed in biological duplicate.

## Results

### Effect of agitation speed, inoculum concentration and culture medium concentration on the biomass production of *Bifidobacterium adolescentis*

**FIGURE 1:**
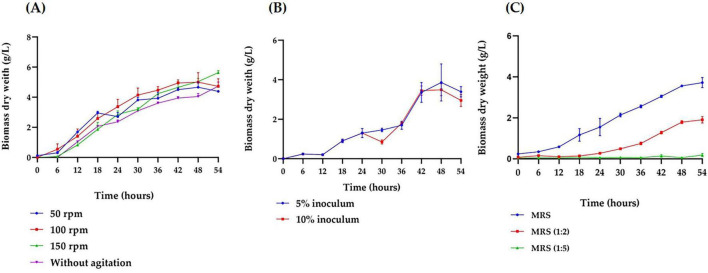
Growth kinetics of *Bifidobacterium adolescentis* under varying microculture conditions. **(A)** Effect of agitation speed (50, 100, and 150 rpm) on the growth in a 5 mL MRS broth medium under anaerobic conditions, with a 5% inoculum, at 37 °C for 54 h. **(B)** Effect of inoculum concentration (5% and 10%) on the growth in a 25 mL MRS broth medium under anaerobic conditions, at 37 °C, 150 rpm, over 54 h. **(C)** Effect of medium concentration (undiluted MRS, 1:2 diluted MRS, and 1:5 diluted MRS) on the growth of in a 25 mL culture under anaerobic conditions, with a 5% inoculum, at 37 °C, 150 rpm, over 54 h. Data points represent the mean from three independent experiments, with error bars indicating standard deviation. Statistical analysis was conducted to assess significant differences between groups, with the appropriate test (parametric or non-parametric) chosen based on data distribution.

The growth of *B. adolescentis* under various conditions was evaluated in microculture. Agitation speed significantly influenced bacterial growth, with higher speeds resulting in increased biomass production (*p* < 0.001) compared to static cultures. At 150 rpm, the bacteria remained in the exponential phase, resulting in an increase of 0.87 g/L in biomass concentration–an 84.64% rise compared to cultures without agitation (Figure 1A). Then, inoculum concentration was evaluated at 5% (v/v) and 10% (v/v), showing similar biomass production throughout most of the cultivation period. However, a significant increase (*p* < 0.001) in biomass was observed at hour 30 with the 5% inoculum compared to the 10% (Figure 1B). Finally, the concentration of the culture medium had a significant effect on bacterial growth (*p* < 0.001). The MRS medium diluted 1:5 did not support *B. adolescentis* growth, while the 1:2 diluted medium allowed for an exponential growth phase, though with lower biomass levels compared to the undiluted medium. The undiluted MRS medium yielded the highest biomass concentration (3.72 g/L), 51.33% higher than in the 1/2 diluted medium (Figure 1C). See statistical analysis in Supplementary Table.

### Evaluation of *B. adolescentis* growth in a 3 L bioreactor

**FIGURE 2:**
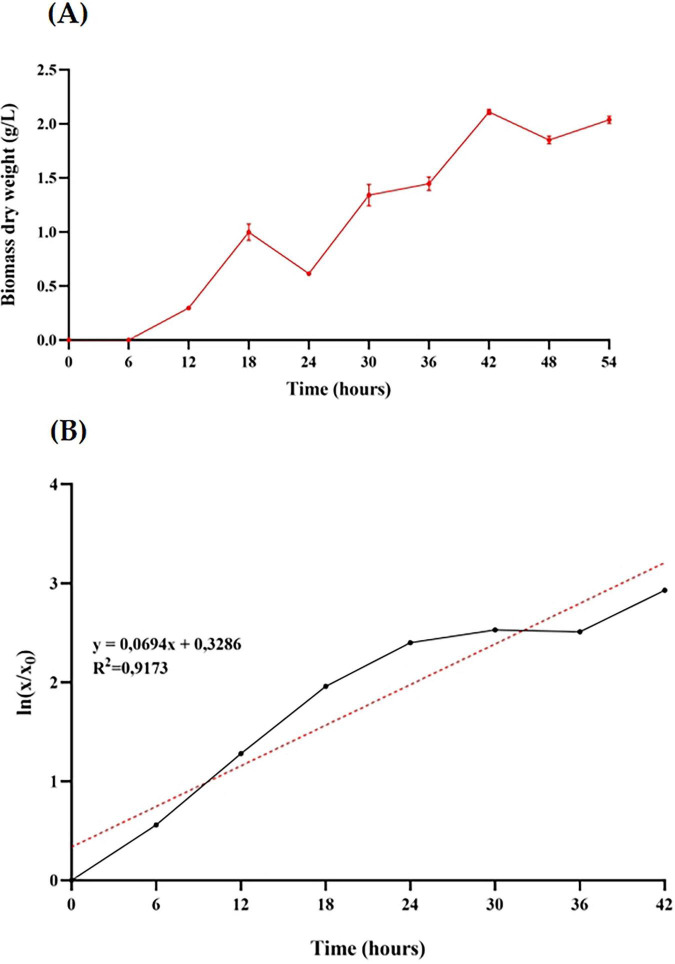
Growth characterization of *Bifidobacterium adolescentis* in a lab-scale anaerobic bioreactor. **(A)** Biomass dry weight (g/L) over time during batch fermentation in a 3 L bioreactor with 1.5 L MRS medium, inoculated at 5% (v/v) and maintained at 37 °C with 200 rpm agitation under anaerobic conditions for 54 h. Data points represent the mean ± standard deviation of three independent measurements. **(B)** First-order kinetic model fitted to the biomass growth data from panel A. The red dotted line indicates the linear regression trend line, with the corresponding equation and R^2^ value shown.

After identifying the most favorable growth conditions (inoculum size 5%, undiluted MRS medium, 37 °C, 200 rpm) for *B. adolescentis* in microculture, these parameters were applied to scaled-up cultivation in a 3 L anaerobic bioreactor (1.5 L working volume) to enhance biomass yield and monitor growth dynamics. As shown in Figure 2A, the culture exhibited a typical batch growth curve, with exponential growth occurring between 6 and 42 h and reaching a maximum biomass concentration of 2.109 g/L. A reduction in growth rate beyond 42 h indicated entry into the stationary phase.

To describe the growth kinetics, a first-order model was applied to the biomass data (Figure 2B). The linear regression of the natural logarithm of biomass over time yielded a coefficient of determination (R^2^) of 0.9173, with a specific growth rate (μ) of 0.069 h^–1^ and a corresponding doubling time of approximately 10 h. These results confirm the consistency of the exponential growth phase under anaerobic, controlled bioreactor conditions.

Following the improvement of growth conditions for *B. adolescentis* in a bioreactor setting, three batches of BaP were produced under nominally identical conditions. For downstream biological assays, Batch 2 was randomly selected from the three BaP production runs. Although some fluctuations were observed in gas injection during anaerobic condition adjustment across batches–particularly a transient pressure increase in Batch 1–Batch 2 maintained appropriate culture conditions and yielded 2.3 mg/mL of total protein, suitable for functional evaluation.

Batch-to-batch variability in protein yield was observed despite standardized conditions. This variation may reflect differences in PEG precipitation efficiency, harvest timing relative to the growth phase, or fluctuations in dissolved gas composition. To improve comparability, a normalized protein/biomass ratio was calculated. Native PAGE followed by silver staining confirmed the presence of high-molecular-weight proteins in all batches, consistent with prior preparations (Supplementary Figure 2).

### Toxicity of BaP: effects on cell viability and death markers related to apoptotic/necrotic responses

**FIGURE 3:**
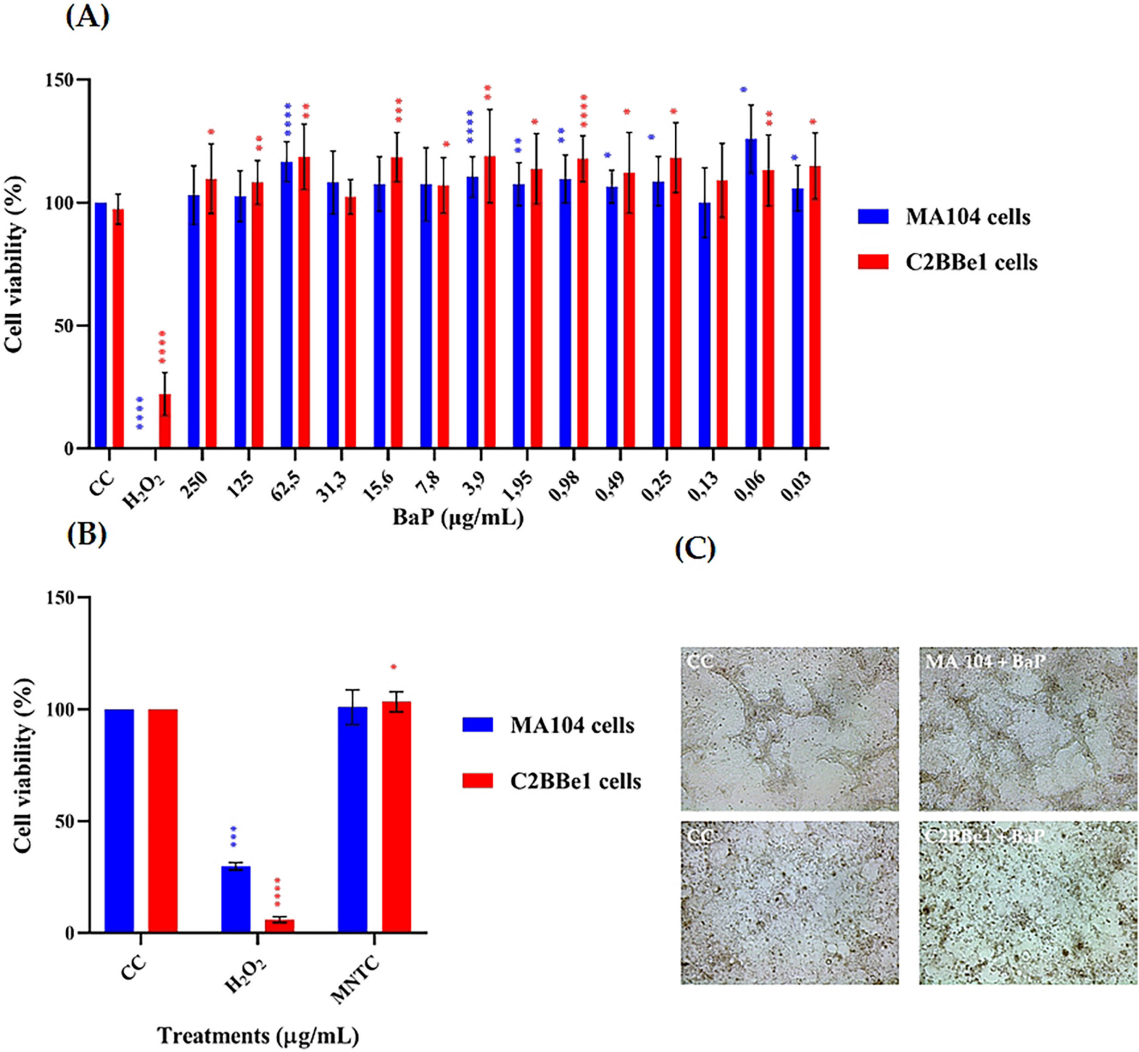
Effect of single and multiple doses of BaP on cell viability percentage (MTT colorimetric assay, Abs 540 nm) and morphology in MA104 and C2BBe1 Cells. **(A)** Effect of a single dose of 14 different concentrations of BaP over 24 h. **(B)** Effect of repeated administration of BaP at the MNTC every 48 h over 14 days. Each bar represents the mean cell viability percentage from three independent experiments; error bars indicate the standard deviation. Comparisons between CC and BaP MNTC and H_2_O_2_ were analyzed using the Mann-Whitney U test at a 95% confidence level. Significance levels are indicated as follows: *p ≤ 0.05, **p ≤ 0.01, ***p ≤ 0.001, ****p ≤ 0.0001. CC, cell control; H_2_O_2_, positive control. **(C)** Photographs of MA104 and C2BBe1 cells, either untreated or treated with BaP at the MNTC, were captured at 100× magnification using an Olympus CKX-41 microscope (Olympus Corporation, Tokyo, Japan) equipped with a Moticam camera and MoticImages software. The images display classic epithelial cells morphology in monolayers and highlight an increase in treated cell clusters.

Cytotoxicity was assessed using the MTT assay for both single and repeated doses in cells susceptible to RV infection (Figure 3). The results of single-dose exposure in MA104 and C2BBe1 cells are presented in Figure 3A. In MA104 cells, none of the BaP concentrations evaluated caused a significant decrease in cell viability. An increase in cell viability was observed in 8 of the tested concentrations, with the 0.06 μg/mL concentration showing the highest increase, reaching 24%. In C2BBe1 cells, none of the 14 concentrations tested exhibited cytotoxic effects in terms of reduced cell viability. Conversely, 12 of the concentrations significantly increased cell viability compared to the control (*p* < 0.001), with increases up to 22%.

Among all concentrations tested in the MTT assay, 250 μg/mL maintained ≥95% cell viability and was thus defined as the Maximum Non-Toxic Concentration (MNTC). This dose, along with a 50% lower concentration (125 μg/mL), was selected for subsequent assays to assess potential dose-dependent biological effects.

Based on the above results from the single-dose cytotoxicity assay, the Maximum Non-Toxic Concentration (MNTC) of BaP was determined for both MA104 and C2BBe1 cells, and these concentrations were used in repeated-dose cytotoxicity assays. Since 250 μg/mL was the highest concentration tested that maintained ≥95% cell viability, it was selected as the MNTC. To explore potential dose-dependent effects, a second concentration corresponding to 50% of the MNTC (125 μg/mL) was also included in subsequent functional assays. The results indicated no significant reduction in cell viability for either cell line (Figure 3B). However, in C2BBe1 cells, succinate dehydrogenase activity increased significantly by 3.35% (*p* < 0.05) after repeated exposure to BaP. Additionally, some morphological changes, including cell circularization, detachment, and enlargement, were observed in both control and treated groups, with these alterations being more prominent in the MA104 cell line (Figure 3C).

**FIGURE 4:**
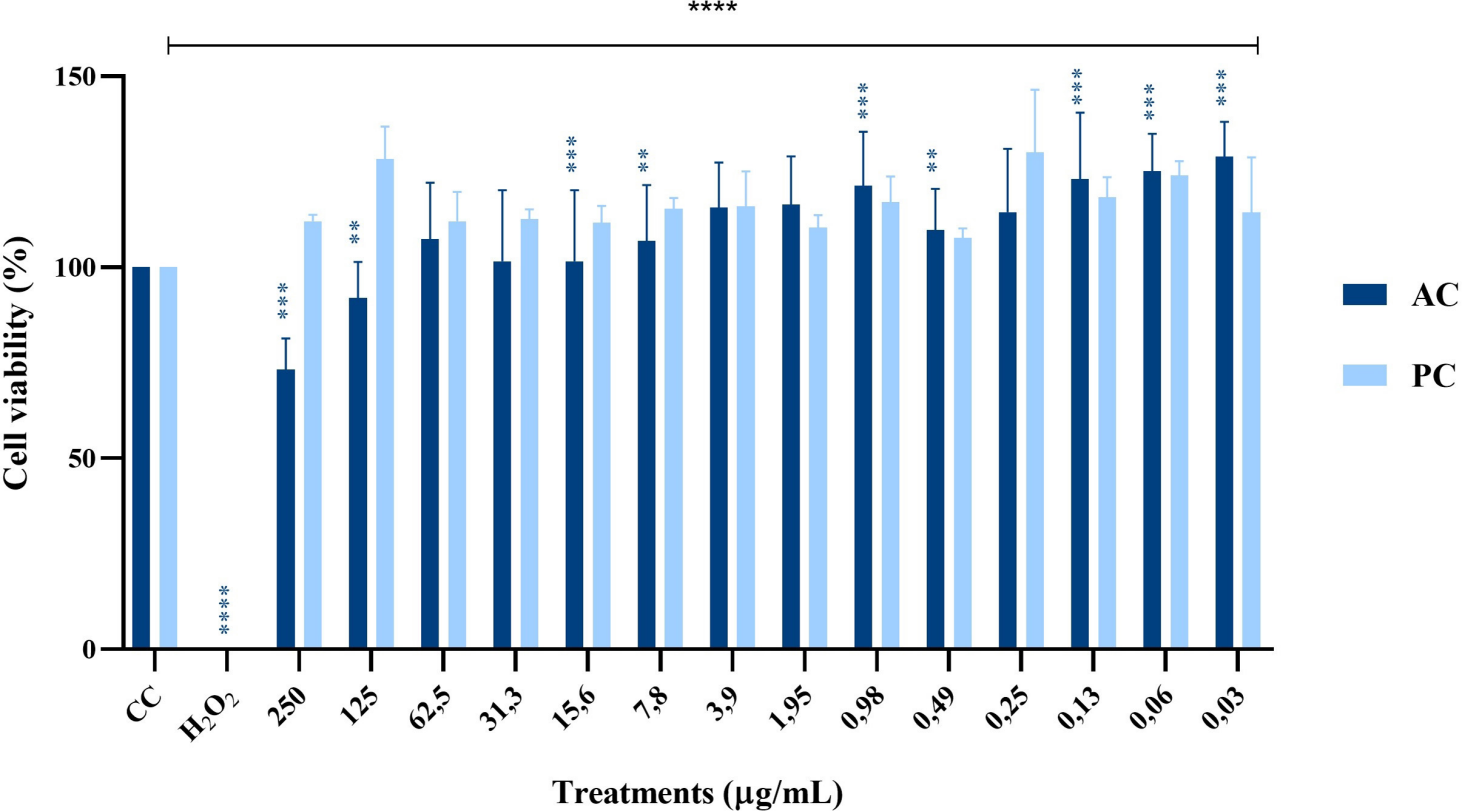
Effect of single doses of 14 different protein concentrations from an Abiotic Control (AC, dark blue) and a Production Control (PC, light blue) on cell viability percentage in MA104 cells, as measured by the MTT colorimetric assay (Abs 540 nm). Each bar represents the mean percentage of cell viability from three independent experiments, with error bars indicating standard deviation. Data were analyzed using an independent samples Mann–Whitney U test. Differences between the evaluated concentrations and the cell control (CC) were significant. Significance levels are indicated as follows: **p ≤ 0.01, ***p ≤ 0.001, ****p ≤ 0.0001. The cell control (CC) corresponds to untreated cells in medium without FBS.

The cytotoxicity evaluation of proteins obtained from the AC and PC is presented in Figure 4. In the AC, protein concentrations of 250 and 125 μg/mL significantly reduced cell viability, while seven of the tested concentrations increased succinate dehydrogenase activity by 9% to 45%. In contrast, none of the 14 BaP concentrations derived from the method by Hernández et al. (2023) showed cytotoxic effects in MA104 cells. However, a significant elevation in succinate dehydrogenase activity was noted, with the 125 μg/mL concentration producing the highest increase, resulting in a viability percentage of 128%.

Regarding the control treatments, the PC–proteins obtained from *B. adolescentis* grown in flasks–exhibited a biological activity profile similar to BaP, with no cytotoxic effects observed across the tested concentrations and a notable increase in succinate dehydrogenase activity, particularly at 125 μg/mL, reaching a viability of 128%. In contrast, the AC, which consisted of sterile MRS medium processed in parallel, showed a cytotoxic profile at higher concentrations, with significant reductions in cell viability observed at 250 and 125 μg/mL. Interestingly, lower concentrations of AC proteins caused a variable increase in succinate dehydrogenase activity ranging from 9% to 45%, suggesting the presence of non-specific components that may alter cellular metabolism. These findings highlight the safety and bioactivity of BaP and PC proteins compared to the non-specific effects seen with the AC.

**FIGURE 5:**
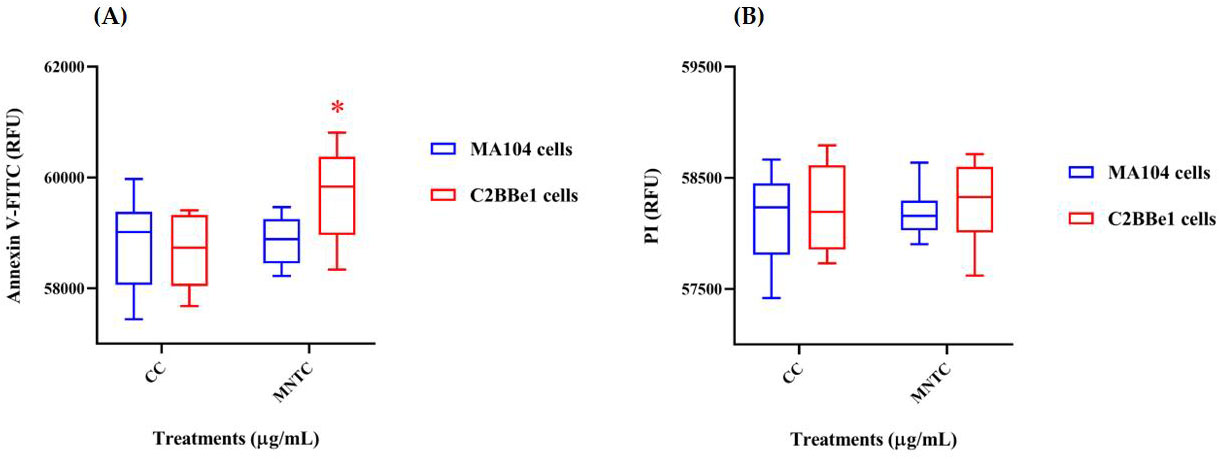
Evaluation of cell death markers in MA104 and C2BBe1 cells after BaP MNTC exposure. Evaluation of cell death markers following exposure to BaP MNTC in MA104 (blue boxes) and C2BBe1 (red boxes) cells for 8 days every 48 h and medium was partially refreshed at each point. **(A)** Phosphatidylserine translocation assessed using Annexin V-FITC (488 nm filter). **(B)** DNA fragmentation evaluated using propidium iodide (PI) fluorochrome (617 nm filter). Boxes represent the mean and interquartile range from three independent experiments with BaP MNTC exposure. Whiskers indicate the maximum and minimum values. Comparisons between the cell control (CC) and BaP MNTC were analyzed using Student’s *t*-test at a 95% confidence level. Significance levels are indicated as follows: *p ≤ 0.05. CC, cell control; H_2_O_2_ positive control.

To gather additional evidence on the potential *in vitro* effects of BaP, a supplementary assay was conducted to assess cytotoxicity by quantifying the expression of cell death markers associated with apoptosis and necrosis (Figure 5). After 8 days of exposure to BaP at the MNTC, administered every 48 h, MA104 cells showed no significant changes in either cell death marker compared to the control (Figure 5A). In contrast, C2BBe1 cells exhibited a significant increase (*p* < 0.05) of approximately 1000 relative fluorescence units (RFU) in phosphatidylserine (PS) translocation, without evidence of DNA fragmentation (Figure 5B).

### Anti-rotavirus activity of BaP

**FIGURE 6:**
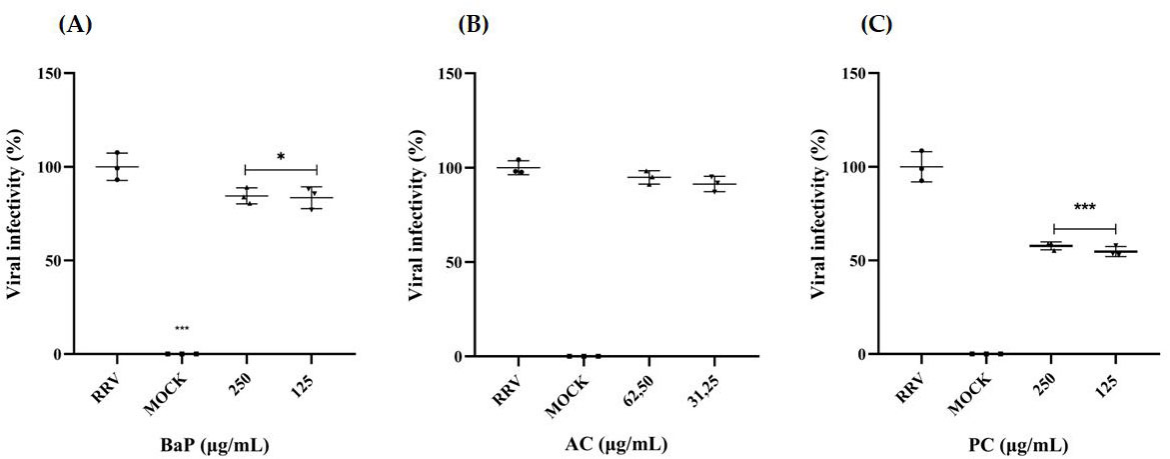
*In vitro* anti-rotavirus activity in MA104 Cells. Rhesus rotavirus (RRV) was co-incubated with two concentrations of **(A)** BaP, **(B)** AC, and **(C)** PC for 1 h at 37°C. MA104 cells were subsequently exposed to the BaP/AC/PC-RRV mixture and incubated for an additional 9 h. Viral infection levels were quantified using immunofluorescence to measure FFU/mL, with infection percentages calculated relative to the positive infection control (RRV). The scatter plot displays mean values with error bars representing standard deviation from three independent samples. Comparisons between the infection positive control (RRV) and BaP treatments were analyzed using Student’s *t*-test at a 95% confidence level, with significance levels indicated as follows: *p ≤ 0.05, ***p ≤ 0.001. RRV, infection positive control; MOCK, uninfected control. Photographs were captured using an Olympus CKX-41 inverted microscope (Olympus Corporation, Tokyo, Japan) equipped with a Lumin Epi-Fluorescence Module and a Moticam camera with MoticImages software, at 100× magnification. The images display Fluorescent Focus Units (FFU) in green, representing RRV-infected cells.

Based on the cytotoxicity evaluation using the MTT assay (Figures 3, 4), the MNTC was determined for BaP, AC, and PC (Figure 6). The MNTC and a half-dose concentration were evaluated for anti-rotavirus activity using a co-incubation approach (virucidal effect) in MA104 cells, followed by quantification of FFU/mL. As shown in Figure 6A, both BaP concentrations (250 and 125 μg/mL) resulted in a significant reduction in rotavirus infection, with decreases of 16% and 17%, respectively. In contrast, the evaluation of the antiviral activity of the proteins obtained from the AC (Figure 6B) showed no reduction in infection percentage at any of the concentrations tested. For PC proteins, both evaluated concentrations significantly reduced the infection percentage by 42% and 45%, respectively (Figure 6C).

### Effect of BaP on cytoskeletal architecture in human intestinal cells post-rotavirus infection.

**FIGURE 7:**
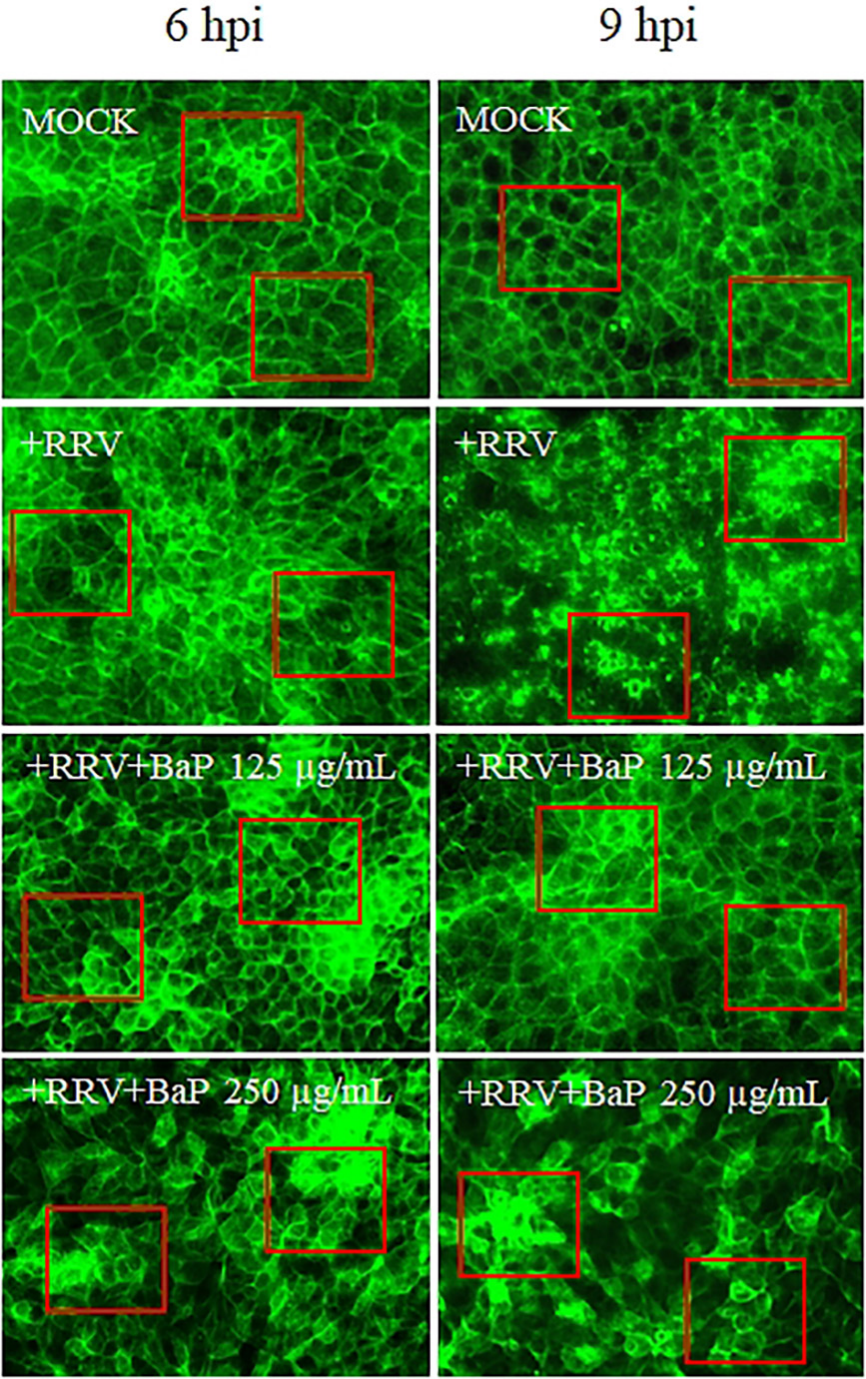
Effect of BaP at MNTC and half-maximal concentration on cytoskeletal architecture in RV-Infected C2BBe1 cells at 6 and 9 hpi. Photomicrographs display cell morphology and distribution in uninfected control cells (MOCK), infected untreated control cells (+RRV), and each BaP treatment condition. Green fluorescence (F-actin) indicates cytoskeletal organization. Red boxes highlight representative areas where morphological changes and differences in F-actin organization are most evident.

The impact of BaP on the cytoskeletal structure of C2BBe1 cells following rotavirus (RV) infection was evaluated at the Maximum Non-Toxic Concentration (MNTC) and a half-dose concentration (250 and 125 μg/mL) (Figure 7). Cells treated with 250 μg/mL BaP at 6- and 9-h post-infection (hpi: hours post-infection) exhibited distinct morphological changes compared to uninfected controls. While untreated infected cells exhibited pronounced cytoskeletal reorganization, resulting in a loss of cell morphology with predominant rounding at 9 hpi, BaP-treated cells showed a morphology similar to the MOCK control, with a higher concentration of F-actin observed at both 6 and 9 hpi when treated with 250 μg/mL. Additionally, cells treated with 125 μg/mL of BaP displayed a more homogeneous morphology, more closely resembling the untreated control compared to those treated with 250 μg/mL, an effect evident at both 6 and 9 hpi.

## Discussion

Scaling up the production of probiotic-derived bioactive compounds poses a significant biotechnological challenge, especially when aiming to preserve biological activity and ensure reproducibility. In this study, we established a laboratory-scale bioprocess to improve the cultivation of *Bifidobacterium adolescentis* and the downstream isolation of protein-based postbiotics (BaP) in a bioreactor system. Our findings contribute to the development of functional postbiotic candidates for gastrointestinal viral disease management, specifically rotavirus (RV) infections, which continue to represent a global burden with limited antiviral therapies.

Agitation rate was identified as a key parameter influencing biomass production. In microcultures, increased agitation (150 rpm) promoted a more favorable environment for *B. adolescentis* growth, likely by enhancing mass and heat transfer, as previously reported for other Bifidobacteria (Hsu et al., 2007). In contrast, cultures grown in a bioreactor equipped with Rushton turbines at 200 rpm produced significantly lower biomass (2.109 g/L), despite the higher agitation speed. This discrepancy may be attributed to the distinct hydrodynamic environments: while orbital shaking minimizes shear stress, Rushton turbines create intense turbulent mixing and high shear forces, which can negatively impact microbial growth and viability (Arnaud et al., 1993; Wang et al., 2020). Furthermore, the favorable microenvironment of small-scale cultures may incidentally support better growth by providing a higher gas-liquid interface and improved nutrient diffusion (Kram and Finkel, 2014).

The impact of medium concentration was also critical. Undiluted MRS medium yielded significantly more biomass than diluted formulations, which is in line with studies demonstrating the dependence of *Bifidobacterium* spp. on nitrogen-rich substrates and milk-derived peptides (Gomes et al., 1998; Cui et al., 2022). Although inoculum size did not significantly affect biomass at late time points, transient advantages were noted at specific intervals, consistent with previous findings showing the influence of inoculum size on lag phase duration and metabolic adaptation (Augustin et al., 2000; Robinson et al., 2001).

Although the three BaP production batches were conducted under nominally identical bioreactor conditions, a marked variability in protein yield was observed (486–2571 μg/mL). The reduced yield in Batch 1 was likely due to a transient pressure increase during the anaerobic stabilization phase, caused by delayed adjustment of gas flow rates using rotameters, which are inherently imprecise. Once gas calibration was corrected, Batches 2 and 3 exhibited more stable environmental conditions, although minor fluctuations in gas injection persisted. Batch 2, randomly selected for downstream biological assays prior to identifying these deviations, yielded 2.3 mg/mL and retained functional activity and safety *in vitro*. This variability highlights common limitations in early-stage bioprocess development using manual gas control. In contrast, industrial-scale bioreactors rely on mass flow controllers (MFCs), which enable precise and reproducible gas delivery, improving process stability and batch-to-batch consistency. Therefore, while some variability was observed under experimental conditions, these deviations are technically resolvable and do not compromise the platform’s scalability or translational potential.

Despite this variability, the bioreactor enabled greater control over anaerobic conditions, pH, and process reproducibility–key parameters for industrial applications. Moreover, a preliminary cost-efficiency estimate suggests a ∼25% reduction in production cost per milligram of protein under bioreactor conditions due to lower manual labor, increased batch volume, and reduced risk of contamination. These findings support the feasibility of scaling BaP production for functional postbiotic formulations using controlled fermentation systems.

In terms of antiviral activity, BaP significantly reduced rotavirus infectivity by 16%–17% in MA104 cells, confirming its biological functionality. However, the PC, derived from *B. adolescentis* cultured in flasks, exhibited higher antiviral activity (42%–45%). This discrepancy may be attributed to differences in culture conditions such as surface-to-volume ratio, oxygen availability, and shear stress, which can influence the expression and secretion of bioactive proteins. In bioreactors, the higher turbulence and altered gas transfer dynamics may affect protein yield or stability compared to flask conditions, which are typically gentler and more static (Grześkowiak et al., 2011; Wang et al., 2020). Supplementary Figure 2 shows the native PAGE analysis of BaP preparations, confirming the presence and reproducibility of protein bands across production batches. While flask culture may favor optimal bioactivity in small-scale settings, bioreactor-based production offers advantages in control, scalability, and reproducibility. These findings support the continued development of BaP as a safe and scalable postbiotic candidate with antiviral potential against rotavirus, particularly in the absence of specific therapeutics and in the context of limited vaccine efficacy in certain populations (Donato et al., 2021).

Postbiotic proteins obtained from the improved bioreactor process demonstrated low cytotoxicity in both MA104 and C2BBe1 cell lines, even after repeated exposure over 14 days. Several BaP concentrations increased cell viability, likely due to mild bioactivation or proliferative signaling through pathways such as MAPK/ERK or PI3K/Akt, as reported in intestinal epithelial models (Yan et al., 2007; Liu et al., 2020; Abdulqadir et al., 2023). This safety profile, together with the absence of DNA fragmentation and only mild phosphatidylserine (PS) externalization, indicates that BaP do not induce cytopathic stress. The PS externalization pattern observed in C2BBe1 cells after prolonged BaP exposure, without DNA fragmentation, is consistent with reversible membrane asymmetry rather than irreversible apoptosis (Balasubramanian et al., 2007). Similar reversible phenomena have been linked to probiotic-derived proteins such as p40 and p75, which activate PI3K/Akt pro-survival signaling in intestinal epithelial cells (Yan et al., 2007), potentially contributing to the preservation of F-actin cytoskeletal architecture observed in BaP-treated cultures.

To address the translational relevance of BaP’s bioactivity, we complemented our analysis in MA104 cells with additional assays in the human intestinal epithelial cell line C2BBe1, a brush border–forming subclone of Caco-2 cells. These cells exhibit a well-developed apical cytoskeleton, organized microvilli, and tight junctions, closely mimicking the morphological and functional features of mature enterocytes (Peterson and Mooseker, 1992). Although direct antiviral activity was only quantified in MA104 cells, we evaluated the protective effect of BaP on cytoskeletal architecture in C2BBe1 cells following rotavirus infection. BaP treatment preserved F-actin organization and epithelial morphology post-infection, suggesting a role in maintaining epithelial integrity during viral insult. While MA104 cells remain the gold standard for rotavirus infectivity assays due to their high permissiveness (Estes and Cohen, 1989; Arnold et al., 2009), the inclusion of C2BBe1-based morphological assays reinforces the relevance of BaP’s protective effects in a physiologically representative human intestinal model.

Interestingly, the intermediate concentration of BaP (125 μg/mL) showed superior preservation of cytoskeletal integrity in C2BBe1 cells compared to the higher dose (250 μg/mL). This inverse dose–response may stem from several concurrent mechanisms. One possibility is partial aggregation of bioactive proteins at higher concentrations, potentially masking functional domains or limiting their interaction with cellular targets (Wang, 2005; Cromwell et al., 2006). Additionally, PEG molecules can modulate protein conformation and dynamics through crowding or soft non-covalent interactions, and even moderate concentrations of PEG are known to reduce protein solubility or mask binding sites (Liebau et al., 2024). Finally, the pattern may reflect a biphasic or hormetic response, where intermediate concentrations elicit maximal benefit, while higher levels result in diminished or counter-regulatory effects (Calabrese and Baldwin, 2003). Together, these findings underscore the relevance of dose optimization in postbiotic applications and support future studies employing finer concentration gradients and refined protein precipitation methods to ensure consistent and effective biological responses.

Although the antiviral and cytoprotective effects of BaP may be slightly attenuated under scaled-up bioreactor conditions, key functional properties, including F-actin stabilization, are retained. Furthermore, *in vivo* studies are needed to define the optimal dosing window that maximizes BaP bioactivity while minimizing unnecessary cellular remodeling. This approach has been emphasized by recent consensus articles and reviews on postbiotic translational research, which underscore the importance of determining effective and safe dose ranges through rigorous preclinical evaluation (Salminen et al., 2022; Asefa et al., 2025).

The mechanisms by which BaP exerts cytoskeletal protection and antiviral effects remain to be fully elucidated. One plausible hypothesis is that bioactive components within BaP interact with epithelial pattern recognition receptors (PRRs), such as Toll-like receptors (TLRs), particularly TLR2 and TLR4. These interactions can activate signaling cascades that modulate cytoskeletal remodeling and epithelial integrity (Tsilingiri et al., 2012; Aguilar-Toalá et al., 2018). Vesicles derived from probiotic bacteria have been shown to enhance intestinal barrier function by increasing the expression of tight junction proteins like ZO-1 and occludin, and by suppressing NF-κB-mediated inflammation (Domínguez Rubio et al., 2022; Zhang et al., 2024). In the context of rotavirus infection, BaP may counteract NSP4-induced calcium dysregulation–a known trigger of cytoskeletal collapse (Hyser et al., 2010; Desselberger, 2014). In our previous study (Olaya Galán et al., 2016), we showed that proteins secreted by *B. adolescentis* reduced NSP4 production in rotavirus-infected cells, suggesting a direct antiviral effect and potential attenuation of virus-induced cytoskeletal disruption. On the other hand, our research group and collaborators (manuscript in preparation) have shown by surface plasmon resonance that PEG-precipitated postbiotic proteins from *B. adolescentis* bind directly to infectious rotavirus particles, with PEGylation affecting affinity and kinetics. This direct interaction may contribute to BaP’s virucidal effect, alongside its barrier-protective and immunomodulatory properties.

Characterization of PEG-precipitated proteins presents analytical challenges due to aggregation, heterogeneity, and interference from residual PEG, which can impair chromatographic resolution and hinder detection in mass spectrometry. These issues have been well documented in PEGylated protein processing (Ramos-de-la-Peña and Aguilar, 2020) and are partly attributable to non-specific PEG–protein interactions (Zheng et al., 2007). In order to obtain reliable proteomic profiles, sample preparation protocols must incorporate additional purification steps such as dialysis, ultrafiltration, or PEG-specific scavenging methods. In the present study, although our main focus was on functional evaluation, we are currently optimizing these protocols to facilitate accurate LC-MS/MS-based identification of the key bioactive proteins. This will strengthen the mechanistic understanding of BaP and support its future formulation and scale-up.

Altogether, the postbiotic proteins derived from *B. adolescentis* exhibit a promising combination of safety, epithelial-protective effects, and moderate antiviral activity under controlled bioprocessing conditions. While further improvements in yield and activity may be achievable through advanced bioprocess engineering or formulation strategies, the current data validate BaP as a functional candidate for oral interventions against gastrointestinal infections. Future work should focus on elucidating the molecular identity of active components and evaluating their performance in complex biological systems. These efforts will help define the mechanisms of action and therapeutic potential of BaP, supporting its translation into a safe, scalable, and effective postbiotic intervention for gut health.

Beyond antimicrobial and structural effects, multiple studies have demonstrated that postbiotics derived from *Bifidobacterium* and *Lactobacillus* strains are capable of modulating proinflammatory cytokines–such as IL-6, IL-1β, TNF-α, and IL-8–with some also promoting regulatory cytokines like IL-10 in intestinal epithelial cells under inflammatory or infectious conditions (Tsilingiri et al., 2012; Aguilar-Toalá et al., 2018). For instance, *Bifidobacterium*-derived postbiotic fractions have been shown to reduce IL-8 and IL-1β secretion in polarized human intestinal tissue models (Tsilingiri et al., 2012), and extensive reviews highlight these immunomodulatory effects in the context of gut epithelial function and homeostasis (Aguilar-Toalá et al., 2018). Such findings reinforce the possibility that BaP may confer not only cytoskeletal protective effects but also immunological modulation in rotavirus-infected epithelium. Future work should therefore include evaluation of cytokine responses, particularly IL-1β, TNF-α, IL-6, IL-8, and IL-10, to elucidate BaP’s immunoprotective mechanisms.

## Conclusion

This study demonstrates that postbiotic proteins from *Bifidobacterium adolescentis* can be produced under bioreactor conditions while retaining key biological activities. BaP preparations were non-cytotoxic, showed moderate anti-rotavirus effects, and preserved epithelial structure. These results highlight BaP’s potential as a scalable and functional postbiotic for gut health applications. Further research should define the active components and validate efficacy *in vivo*.

## Data Availability

The original contributions presented in this study are included in this article/Supplementary material, further inquiries can be directed to the corresponding author.
