# Supplementary material for: Lab-scale production of postbiotic proteins from Bifidobacterium adolescentis with antiviral and epithelial-protective properties
Source: Front Microbiol. 2025 Oct 1;16:1646082. doi: 10.3389/fmicb.2025.1646082 (PMC12521213; doi:10.3389/fmicb.2025.1646082)
Supplement: Supplementary file 1 [file Data_Sheet_1.docx]

Supplementary Material

# Supplementary Figures


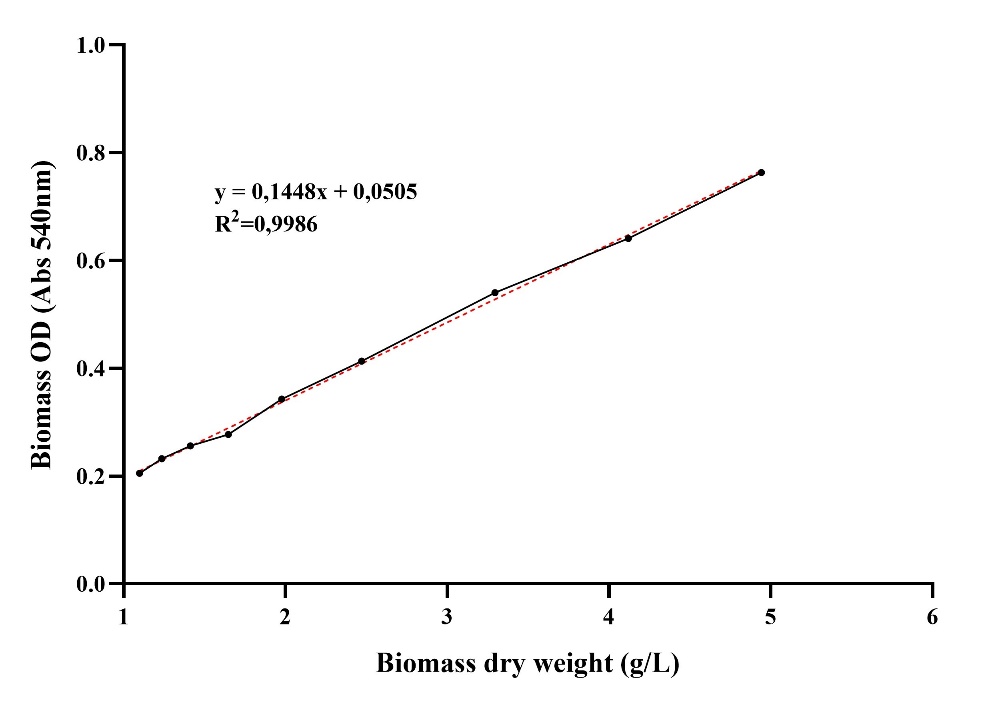


**Supplementary Figure 1.** Calibration curve correlating absorbance with biomass dry weight (g/L). The black points represent experimental measurements, and the red line corresponds to the linear regression model fitted to the data. This calibration was used to estimate biomass concentration in subsequent growth experiments.


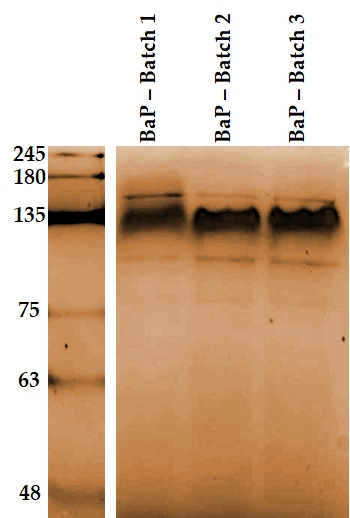


**Supplementary Figure 2.** Native polyacrylamide gel electrophoresis (7.5% PAGE) showing protein profiles of BaP preparations obtained from three production batches. From right to left: molecular weight marker (kDa), and BaP protein samples from batches 1, 2, and 3. Each lane was loaded with 100 ng of total protein. Silver nitrate staining was used for protein visualization.

# Supplementary Table

*p*-values and overall statistical summary from the analysis of *B. adolescentis* growth under varying culture conditions. For the agitation speed experiment, statistical comparisons were performed between each agitation condition and the static (non-agitated) control. For the inoculum concentration experiment, differences between the 5% and 10% inoculum levels were analyzed. For the medium concentration experiment, statistical comparisons were conducted between each diluted MRS formulation and the undiluted MRS control.
